# Antihypertensive Drug Use and COVID‐19 Disease Severity in Hospitalized US Veterans: A Retrospective Cohort Study

**DOI:** 10.1111/jch.70021

**Published:** 2025-02-24

**Authors:** Merodean Huntsman, Jessica L. Marquez, Gregory J. Stoddard, Guo Wei, Aaron J. Miller, Jayant P. Agarwal, Sujee Jeyapalina

**Affiliations:** ^1^ Division of Plastic and Reconstructive Surgery Department of Surgery University of Utah School of Medicine Salt Lake City Utah USA; ^2^ George E. Wahlen Department of Veterans Affairs Medical Center Salt Lake City Utah USA; ^3^ Division of Epidemiology Department of Internal Medicine University of Utah School of Medicine Salt Lake City Utah USA

**Keywords:** antihypertensive medications, COVID‐19, diuretics, VA COVID‐19 shared resources, veterans

## Abstract

This research investigated associations between hypertensive patients with COVID‐19 who did and did not use specific antihypertensive medications and had incurred hospital ventilation/death in the US Veterans Hospital System. This study included de‐identified medical records of 572 994 hypertensive US veterans who were diagnosed with COVID‐19 and hospitalized between March 1, 2020, and February 28, 2022, with a 60‐day follow‐up period. Mechanical ventilation and/or death within 60 days of COVID‐19 hospitalization served as the outcome variables. On multivariable analysis, CCB use was associated with a 9% increased risk of mechanical ventilation, while ACE inhibitors (HR: 0.90), alpha blockers (HR: 0.92), and CCB (HR: 0.93) users were associated with a significantly decreased risk of death. Additional multivariable analysis on those with and without additional comorbidities, such as chronic kidney disease (CKD), heart failure, and diabetes, revealed that in those with at least one additional comorbidity, CCB uses led to a 9% increased deleterious risk for ventilation. In contrast, the use of ACE inhibitors (HR: 0.86), alpha blockers (HR: 0.92), and CCB (HR: 0.93) demonstrated a moderate protective effect against mortality. Lastly, in hypertensive veterans without any additional comorbidities, there was a lack of significant association between hypertensive medication usage and mechanical ventilation and/or death. In summary, although CCB use was associated with an increased risk of requiring mechanical ventilation, it also demonstrated a protective effect against mortality. At the same time, ACEI, alpha blocker, and CCB usage led to a significantly decreased risk for death within all hypertensive hospitalized COVID‐19 veteran cohorts.

## 1 Introduction

On March 11, 2020, the World Health Organization declared Coronavirus Disease 2019 (COVID‐19) as a global pandemic. While most infected people experience mild symptoms, some patients encounter severe symptoms, resulting in pneumonia, acute respiratory distress syndrome (ARDS), multi‐organ dysfunction, and even death [1]. Against the backdrop of global spread, clinicians and scientists have begun to question why some individuals suffer severe complications while others develop only a mild infection or remain asymptomatic. A comprehensive analysis of CDC data shows association between certain comorbidities, such as hypertension, cardiovascular disease, diabetes, obesity, respiratory disease, liver disease, and chronic kidney disease (CKD), and markedly increasing illness severity, hospitalization and even death [2]. Among all reported comorbidities, the severity of illness resulting from COVID‐19 infection is mainly skewed toward hypertensive individuals, who experience a 2.5‐fold increased risk of symptom severity [3, 4].

In the early stages of the pandemic, scholars postulated that the reason for the high proportion of case fatality amongst hypertension patients could potentially be attributed to the over‐expression of angiotensin‐converting enzyme 2 (ACE2) receptors and related mechanisms [3]. It was suggested that as the spike protein of the SARS‐CoV‐2 virus uses ACE2 to gain access into the cells, overexpressed ACE2 in hypertension patients allows a higher rate of viral cellular infiltration and, therefore, more severe symptoms [5]. Because the ACE2 receptors play a crucial role in the counter‐regulation of blood pressure, the expression of ACE2 within the patient population with cardiovascular diseases, including hypertension, has been hypothesized to be differentially modulated with exposure to certain classes of antihypertensive medication [6, 7]. Although no clear data support the association of angiotensin‐converting enzyme inhibitors (ACEIs) or angiotensin II blockers (ARBs) with increased disease severity and mortality [8, 9], some early literature questioned their safety in patients with COVID‐19 [10]. This has led to uncertainty among hypertensive patients regarding their medication use. Thus, the effect of ACEIs and ARBs on COVID‐19 severity and death in hypertension patients has been a topic of interest in recent literature [10]. Meanwhile, there has been relatively little reported on other common classes of hypertension medications, such as calcium channel blockers (CCBs), beta blockers, alpha blockers, and diuretics in hypertensive patients. Of the studies that did include the aforementioned medication classes, the majority were highly underpowered to make any conclusions regarding their continuous use [8, 11–25].

As such, this study was designed to examine the associations between all six classes of hypertension medications and COVID‐19 severity as measured by mechanical ventilation usage and in‐hospital death amongst COVID‐19‐positive US veterans with hypertension.

## 2 Methods

### 2.1 Study Design and Data Resources

This retrospective cohort study utilized electronic medical records of hypertensive US veterans diagnosed with COVID‐19 and hospitalized between March 1, 2020, and February 28, 2022, within the nationwide Department of Veterans Hospital Administration (VHA). A selection of information related to SARS‐CoV‐2‐tested veterans, such as COVID‐19 infection index date, type of test, death or hospital discharge, ventilation, comorbidities, and medication use, was collected from the COVID‐19 Shared Data Resources. This shared data resource contains information on veterans who were diagnosed with COVID‐19 within VHA collected from the VHA's Corporate Data Warehouse (CDW) [26]. Within the COVID‐19 Shared Data Resources, the index date was defined as the first positive SARS‐CoV‐2 test date or the hospitalization admission date if veterans received care 15 days before the positive test date. Patient's hypertensive medication usage within 90 days prior to the index date was used for analysis. Severity was defined as mechanical ventilator use or death within 60 days of hospitalization/index date.

### 2.2 Ethical Statement and Institutional Approvals

The Institutional Review Board determined this study (# 00133238) to be exempt and waived the requirement for informed consent due to the de‐identified nature of the data. The COVID‐19 Shared Data Resources were accessed and analyzed via the VA informatics and computing infrastructure server (VINCI), ensuring veterans' privacy and data security.

### 2.3 Inclusion Criteria

For the present study, inclusion criteria included patients who were (1) active users of the VA hospital system, (2) veterans aged 18 years and older, (3) hospitalized COVID‐19‐positive veterans with an index date between March 1, 2020, and February 28, 2022, with a minimum of 60‐day follow‐up, and (4) diagnosed with hypertension at least 2 years prior to the COVID‐19 index date.

### 2.4 Exposure/Key Study Variable

The variable of interest was the pharmacological exposure to any of six common antihypertensive drugs (ACEIs, ARBs, CCBs, beta blockers, alpha blockers, and diuretics). Veterans were defined as antihypertensive drug users if they used these drugs within 3 months prior to the COVID‐19 index date. Patients who did not use the aforementioned antihypertensive medications during this time period were placed in the zero category of antihypertensive usage.

### 2.5 Covariates

To identify patient characteristics predictive for the clinically measured outcomes, the following regressors were collected and controlled for as potential confounders in the multivariable models: age, gender, race, income, body mass index (BMI), smoking status, alcohol dependence, comorbidities (chronic kidney disease [CKD], chronic obstructive pulmonary disease [COPD], heart failure [HF], diabetes mellitus [DM], coronary artery/heart disease [CAHD]), cancer, HIV, liver disease, sickle cell disease, asthma), and Charlson Comorbidity Index. As the JNC‐8 protocol [27] for first‐ and second‐line antihypertensive treatments for Black Americans differ from that recommended for White Americans and other races, the race categorization and analyses were limited to Black, White, and others.

### 2.6 Statistical Analysis

Cox proportional hazard models were used to test the association between mechanical ventilation and death outcomes and the use of antihypertensive drugs. Hazard ratios (HRs) with 95% confidence intervals (95% CI) and *p* values were reported. All statistical analyses were conducted using R version 4.0.2 (https://www.R‐project.org). Publication‐quality images were obtained using Adobe Photoshop CC 2019 (San Jose, California).

### 2.7 Cox Regression Models

Univariable and multivariable Cox regression analyses were performed, accounting for time to event in days for either mechanical ventilation or death outcomes when a subject had exposure to one of the major classes of antihypertensive medications. Additional multivariable COX regression analyses were performed on this cohort, subdivided into those with and without comorbidities. Significant COVID‐19‐related comorbidities of interest reported by the CDC [2] were considered for inclusion in the analyses. Finally, multivariable Cox models were repeated for a cohort of patients with hypertension only versus with hypertension and one or more comorbidities. Significance tests for the multivariable model were performed at the *p* < 0.05 level.

## 3 Results

Of 572 994 veterans who had tested positive for COVID‐19 with a minimum 60‐day follow‐up during this study period, 305 012 of whom had a history of hypertension prior to COVID‐19 disease onset. Of these COVID‐19‐positive veterans with hypertension, 54 984 (18%) were hospitalized. Patients who met the inclusion criteria were grouped by outcome severity as defined by mechanical ventilation (severe, *n* = 6328 (11.5%)) and in‐hospital death (most severe, *n* = 8373 (15.2%)). This cohort was predominately found to be 96% male, 68% White, and 74% overweight to obese. Approximately 40% of the cohort were 65–74 years of age. Within this cohort, ∼37% never smoked, while ∼46% were former smokers (Table 1).

**TABLE 1:** Demographic and clinical characteristics of COVID‐19‐positive veterans with hypertension.

	COVID‐19‐positive veterans with HTN (*n* = 305 012)	Hospitalized COVID‐19 veterans with HTN (*n* = 54 984)	Mechanical ventilation (*n* = 6328)	In‐hospital death (*n* = 8373)
Mean age, mean ± SD	66 ± 13	70 ± 12	70 ± 10	76 ± 10
Median income, mean ± SD	$59 000 ± $2100	$57 000 ± $2100	$57 000 ± $2000	$57 000 ± $2200
Gender, *n* (%)				
Male	283 663 (93.)	52 582 (95.6)	6100 (96.4)	8182 (97.7)
Female	21 394 (7.0)	2402 (4.4)	228 (3.6)	191 (2.3)
Race, *n* (%)				
Black	69 681 (22.8)	14 911 (27.1)	1797 (28.4)	1927 (23.0)
White	207 907 (68.2)	35 613 (68.4)	3990 (63.1)	5733 (68.5)
Others	27 424 (9.0)	4460(8.1)	541 (8.5)	713 (8.5)
BMI (kg/m^2^), *n* (%)				
Underweight	3414 (1.1)	1615 (2.9)	138 (2.2)	448 (5.4)
Normal weight	44 302 (14.6)	12 659 (23.0)	1139 (18.0)	1139 (18.0)
Overweight	93 640 (30.8)	16 667 (30.3)	1900 (30.0)	2509 (30.0)
Obese	134 239 (44.2)	19 625 (35.7)	2504 (39.6)	2439 (29.2)
Morbidly obese	28 390 (9.3)	4378 (8.0)	645 (10.2)	509 (6.1)
Smoking status, *n* (%)				
Never	112 343 (36.8)	18 062 (32.8)	2080 (32.9)	2532 (30.2)
Current	41 262 (13.5)	7971 (14.5)	811 (12.8)	978 (11.7)
Former	138 860 (45.5)	25 967 (47.2)	3109 (49.1)	4345 (51.9)
Alcohol dependence, *n* (%)	60 096 (19.7)	10 431 (19.0)	981 (15.5)	1075 (12.8)
HTN drugs, *n* (%)				
ACE	76 993 (25.2%)	13 218 (24.0%)	1611 (25.5%)	1797 (21.5%)
ARB	47 062 (15.4%)	8295 (15.1%)	1019 (16.1%)	1231 (14.7%)
Alpha blockers	59 940 (19.7%)	13 285 (24.2%)	1555 (24.6%)	2165 (25.9%)
Beta blockers	86 490 (28,4%)	19 249 (35.0%)	2360 (37.3%)	3105 (37.1%)
CCB	73 585 (24.1%)	14 466 (26.3%)	1834 (29.0%)	2067 (24.7%)
Diuretics	69 620 (22.8%)	15507 (28.2%)	1937 (30.6%)	2521 (30.1%)
Number of antihypertensives used, *n* (%)				
0	108 034 (35.4)	4407 (8.0)	284 (4.5)	692 (8.3)
1	81 397 (26.7)	11 197 (20.4)	938 (14.8)	1602 (19.1)
2	64 967 (21.3)	16 302 (29.6)	1666 (26.3)	2416 (28.9)
3	35 362 (11.6)	14 110 (25.7)	1837 (29.0)	2215 (26.5)
4+	15 252 (5.0)	8968 (16.3)	1603 (25.3)	1448 (17.3)
Comorbidities, *n* (%)				
Asthma	21 819 (7.2)	3972 (7.2)	464 (7.3)	462 (5.5)
CAHD	86 366 (28.3)	21 449 (39.0)	2482 (39.2)	3766 (45.0)
Cancer	51 443 (16.9)	13 362 (24.3)	1575 (24.9)	2471 (29.5)
CKD	61 081 (20.0)	18 324 (33.3)	2242 (35.4)	3579 (42.7)
COPD	61 249 (20.1)	16 754 (30.5)	2006 (31.7)	2866 (34.2)
Diabetes mellitus	141 457 (46.4)	30 492 (55.5)	3824 (60.4)	4820 (57.6)
Heart failure	43 593 (14.3)	14 891 (27.1)	1806 (28.5)	2842 (33.9)
HIV	2182 (0.7)	542 (1.0)	55 (0.9)	53 (0.6)
Liver diseases	25 343 (8.3)	61 66 (11.2)	723 (11.4)	908 (10.8)
Sickle cell disease	537 (0.2)	139 (0.3)	17 (0.3)	20 (0.2)
Number of comorbidities, *n* (%)				
0	64 304 (21.1)	5376 (9.8)	39 (8.5)	555 (6.6)
1	73 942 (24.2)	9558 (17.4)	1089 (17.2)	1161 (13.9)
2	41 561 (13.6)	7545 (13.7)	873 (13.8)	1034 (12.3)
3+	31 150 (41.1)	32 505 (59.1)	3827 (60.5)	5623 (67.2)
Charlson Comorbidity Index, *n* (%)				
0	75 466 (24.7)	6254 (11.4)	664 (10.5)	585 (7.0)
1	65 287 (21.4)	8168 (14.9)	872 (13.8)	961 (11.5)
2	65 287 (21.4)	9007 (16.4)	1056 (16.7)	1210 (14.5)
3+	65 287 (21.4)	31 555 (57.4)	3736 (59.0)	5617 (67.1)

Abbreviations: ACE, angiotensin‐converting enzyme; ARB, angiotensin receptor blocker; BMI, body mass index; CAHD, coronary atherosclerotic heart disease; CCB, calcium channel blocker; CKD, chronic kidney disease; COPD, chronic obstructive pulmonary disease; HIV, human immunodeficiency virus; HTN, hypertension; SD, standard deviation.

Additional clinical characteristics and pharmaceutical usage of this cohort are also given in Table 1, which shows the relative percentages of different classes of antihypertensive users within each symptom severity category. Overall, approximately 23% of this veteran cohort have taken diuretics; however, within the mechanical ventilation and in‐hospital death groups, the percentage increased to ∼30%. Similar trends were also seen for beta and alpha blockers. Notably, nearly 40% of all COVID‐19‐positive hypertensive veterans and over 70% of those requiring hospitalization had been prescribed two or more antihypertension medications. The most common condition within the entire hospitalized cohort included in this study was DM (55.5%), followed by CAHD (39%) and CKD (33.3%). It is worth noting that 59.1% (*n* = 32 505) of hospitalized hypertensive veterans with COVID‐19 had three or more comorbidities. Of the hospitalized veterans, 60.5% who received mechanical ventilation treatment and 67.2% who succumbed to death also had three or more comorbidities. Most (57.4%) hospitalized veterans had a Charlson Comorbidity Index of 3+. It is also worth noting that ∼59% of the veterans who received mechanical ventilation and ∼67% who perished within 60 days had a Charlson Comorbidity Index of 3+.

Cox models were used in univariate and multivariable analyses investigating the effects of the hypertension medications on COVID‐19 symptom severity (Tables 2 and S1).On univariate analyses, male sex, Black race, increased BMI, hypertensive medications (ACEI, ARB, beta blockers, CCB, and diuretics), comorbidities (CKD, COPD, HF, and DM), and increased Charlson Comorbidity Index were associated with increased risk of mechanical ventilation. Advanced age, male sex, White race, underweight, former smoking status, hypertensive medications (alpha blockers, beta blockers, and diuretics), comorbidities (CAHD, cancer, CKD, COPD, HF, and DM), and increased Charlson Comorbidity Index were associated with increased risk of death. A multivariable analysis was then conducted, adjusting for all characteristics in Table 1. After adjusting for the aforementioned potential confounding variates, CCBs (HR: 1.09, 95% CI (1.03–1.16), *p* = 0.002) were independently associated with an increased risk of mechanical ventilation. In contrast, ACEI (HR: 0.90, 95% CI (0.85–0.95), *p* < 0.001), alpha blockers (HR: 0.92, 95% CI (0.87–0.97), *p* = 0.001), and CCB (HR: 0.93, 95% CI (0.88–0.98), *p* = 0.006) were independently associated with a decreased risk for mortality (Table 2).

**TABLE 2:** Univariable and multivariable Cox regression survival analyses of hypertensive medication usage predictive of ventilation and fatality in in‐hospital COVID‐19 and hypertension‐positive veterans.

	Ventilation				Fatality			
	Univariable		Multivariable		Univariable		Multivariable	
	HR (95% CI)	*p* value	HR (95% CI)	*p* value	HR (95% CI)	*p* value	HR (95% CI)	*p* value
Hypertension medications								
ACE inhibitor	1.09 (1.03, 1.15)	**0.005**	1.02 (0.96, 1.08)	0.58	0.86 (0.81, 0.90)	**<0.001**	0.90 (0.85, 0.95)	**<0.001**
ARB	1.08 (1.01, 1.16)	**0.018**	0.99 (0.92, 1.06)	0.72	0.97 (0.91, 1.03)	0.3	0.95 (0.89, 1.01)	0.12
Alpha blocker	1.02 (0.97, 1.08)	0.45	0.96 (0.91, 1.02)	0.19	1.10 (1.05, 1.16)	**<0.001**	0.92 (0.87, 0.97)	**0.001**
Beta blocker	1.11 (1.05, 1.17)	**<0.001**	1.02 (0.97, 1.08)	0.42	1.10 (1.06, 1.15)	**<0.001**	0.99 (0.94, 1.04)	0.69
CCB	1.15 (1.09, 1.22)	**<0.001**	1.09 (1.03, 1.16)	**0.002**	0.91 (0.87, 0.96)	**<0.001**	0.93 (0.88, 0.98)	**0.006**
Diuretics	1.13 (1.07, 1.19)	**<0.001**	1.00 (0.95, 1.07)	0.88	1.11 (1.06, 1.16)	**<0.001**	1.00 (0.95, 1.06)	0.93

Bold Values statistical significance *p* values < 0.05.

**FIGURE 1:**
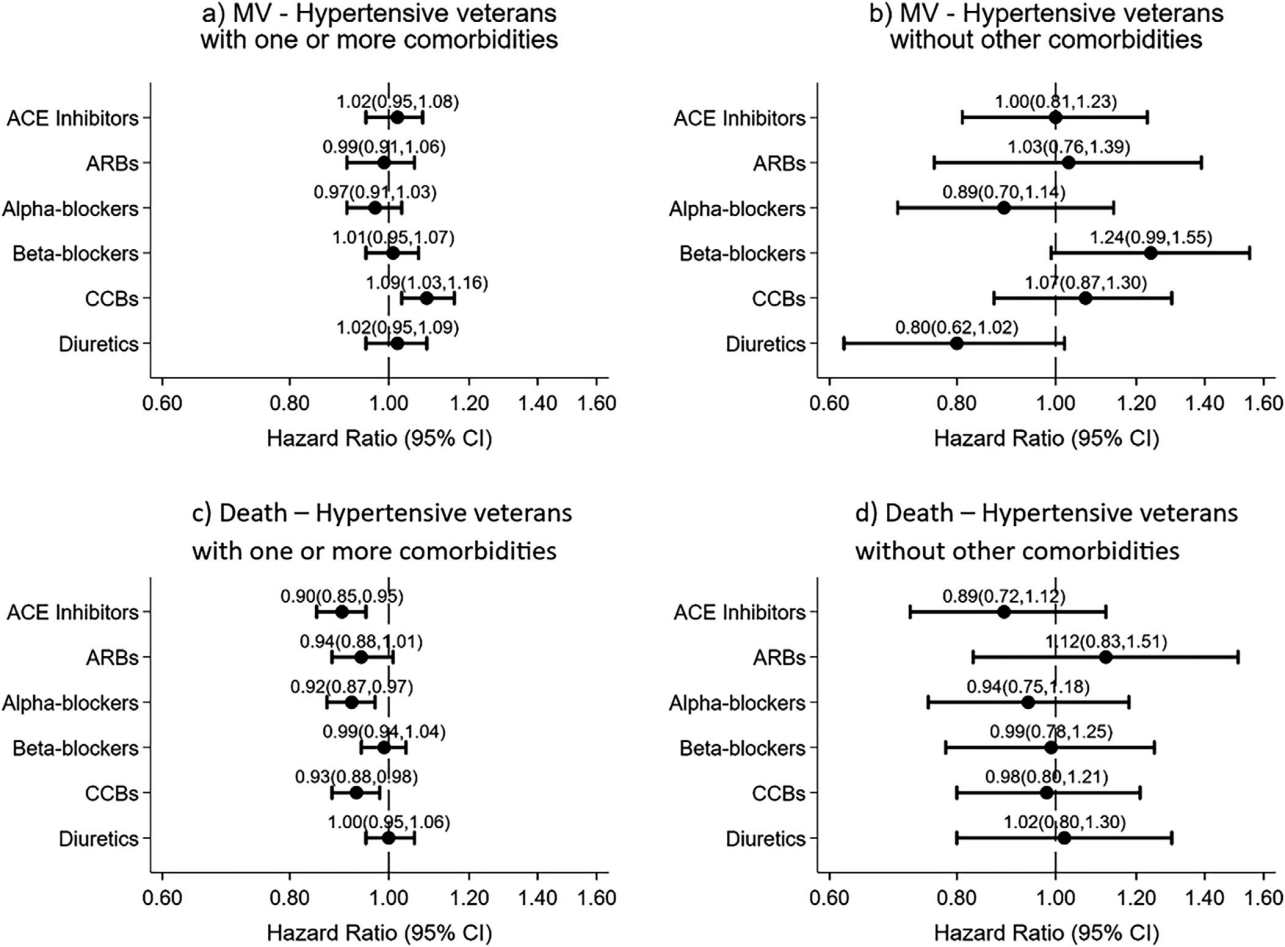
A set of forest plots showing hazard ratios (HR) of COVID‐19‐positive hospitalized veterans with and without additional comorbidities as a function of antihypertensive medication classes. Veterans who required ventilation (a) and (b). Veterans with mortality reports within 60 days of the hospitalization (c) and (d). While (b) and (d) present the HR for hypertensive veterans without additional comorbidities, (a) and (c) show HRs for those with additional co‐morbidities. The following variables were adjusted for age, sex, race, income, BMI, CAHD, cancer, CKD, COPD, heart failure, diabetes, HIV, liver disease, sickle cell anemia, asthma, smoking and alcohol.

To better understand how antihypertensive medications may influence the risk of mechanical ventilation and death in veterans with multiple comorbidities, this cohort was subdivided into the following two groups: those with hypertension in addition to one or more comorbidities and those with hypertension only. Their HR results are graphically represented as forest plots (Figure 1). In the hypertensive with comorbidities group, CCBs increased the risk of mechanical ventilation. ACEI, alpha blockers, and CCBs decreased the risk of death. No medications were associated with a significant increase or decrease in risk of mechanical ventilation or death in the hypertensive‐only group.

## 4 Discussion

This retrospective cohort study analyzed disease severity, as determined by mechanical ventilation or in‐hospital death, and clinical characteristics of over 50,000 hypertensive veterans hospitalized with COVID‐19. Pertinent findings elucidated from the data analyses included the following: (1) Exposures to CCBs increased the risk of mechanical ventilation in patients with hypertension and at least one additional comorbidity, while (2) ACEIs, alpha blockers, and CCBs were independently associated with a significantly reduced risk of in‐hospital mortality (Figure 1). (3) In hypertensive veterans without any additional comorbidities, there was a lack of significant association between hypertensive medication usage and mechanical ventilation and/or death. The data from this study supports some previous antihypertensive drug studies suggesting that pharmacological exposure to ACEIs and CCBs may be associated with a protective effect against COVID‐19 symptom severity (i.e., death), thereby supporting the literature suggesting that hypertensive patients should not discontinue aforementioned medications if infected with SARS‐CoV‐2 virus [8]. It is particularly crucial for hypertensive patients with additional comorbidities to consistently adhere to their prescribed medications.

Importantly, while CCBs were associated with an increased risk of mechanical ventilation, they were also associated with a decreased risk of in‐hospital death. Multiple studies found that the use of CCBs in hospitalized patients with COVID‐19 was associated with a decreased risk for intubation and mortality [28, 29, 30, 31]. However, complications can arise from CCB toxicity and overdose, such as ARDS, which may require mechanical ventilation [32, 33]. This and similar complications resulting from CCB usage may explain their association with increased rates of mechanical ventilation in the Veteran cohort, who has multiple comorbidities, the complexity of their health needs, and the use of multiple medications (polypharmacy) for managing them [34].

Interestingly, alpha blockers were associated with a protective function against death. According to the JNC‐8 recommendations, alpha blockers are a 3^rd^‐line antihypertensive treatment prescribed when hypertension is unable to be controlled by first or second‐line treatments [27]. A possible mechanism for the protective effect of alpha blockers may result from their ability to reduce left ventricular hypertrophy, an independent risk factor for cardiovascular mortality and morbidity, and improve glomerular filtration rate, reducing kidney damage [35]. Given the diversity, complexity, and frequency of confounding comorbidities in hypertensive patients, prospective randomized control or propensity‐matched studies are needed to study the effects of alpha blockers on COVID‐19 severity.

Additional interesting findings included former smokers found to be at higher risk of mortality than current smokers, and both asthma and COPD patients were at decreased risk for mortality. In a study assessing smoking and the risks of COVID‐19 hospitalization, Neira et al. found that patients who were former smokers were more likely to be hospitalized due to COVID‐19. This finding was attributed to the fact that former smokers were, on average, 10 years older than current smokers, and the risk of severe COVID‐19 among former smokers may have been significantly driven by the effect of age and comorbidities [36, 37]. Though we did not find a significant association between smoking status and mechanical ventilation, we did find former smokers (HR: 1.08, 95% CI (1.03–1.14), *p* < 0.001) were at greater risk of mortality when compared to current smokers (Table S1). Although decreased association of asthma (HR: 0.83, 95% CI (0.76–0.92), *p* < 0.001) and COPD (HR: 0.93, 95% CI (0.89–0.98), *p* = 0.01) with in‐hospital death found in our study, there are mixed results found in the literature. For example, one review found an increased association with hospitalization for both conditions. In contrast, another found no association between asthma and in‐hospital mortality [35] but did find an association between COPD and in‐hospital mortality [38]. Interestingly, a study conducted by Calmes et al. concluded that asthma and COPD were not risk factors for ICU admission and death related to SARS‐CoV‐2 infection [39].

Limitations of this study included certain demographic elements of our cohort, which are not representative of the total US population. This hypertensive veteran cohort was disproportionately male, White, and of advanced age. Also, US veterans within the VA hospital system have access to free universal healthcare, which may not be accessible to nonveterans of similar socioeconomic classes. Based on the income data (Table 1), regardless of severity outcomes, veterans were of similar socioeconomic class, earning 57–59K annually. We also did not have data on potential confounding factors such as medication adherence, lifestyle, and so forth. Finally, our death rate was calculated by an all‐cause in‐hospital death, so all deaths might not have been solely due to COVID‐19.

Future studies must investigate the relative protective mechanisms of ACEIs, alpha blockers, and CCBs against mortality. A large‐scale study determining the effect of these medications COVID‐19 severity across multiple nonhypertension cohorts would be beneficial in determining the potential protective/deleterious COVID‐19 symptom severity in diuretic user groups. Additionally, an investigation into the underlying mechanisms between antihypertensive classes and the relative risk of COVID‐19 severity is needed to inform better treatment and direct future clinical care of hypertensive patients.

## 5 Conclusion

In summary, we found that CCB use was associated with higher risks for mechanical ventilation but lower risks for death within a hypertensive hospitalized COVID‐19 veteran cohort. ACEI and alpha blocker usage were also associated with a decreased risk of death. Interestingly, there was a lack of significant association between hypertensive medication usage and mechanical ventilation and/or in‐hospital death in hypertensive patients without additional comorbidities. Additional studies are needed to better elucidate the mechanisms of the above findings.

## Author Contributions

M.H., S.J., and J.P.A. planned the paper. M.H. and S.J. conducted a literature review. G.W. and G.J.S. collected and analyzed data. All authors contributed to the data interpretation and discussion. M.H., J.L.M., A.J.M., and S.J. wrote the paper. All authors edited/approved the manuscript.

## Ethics Statement

The Institutional Review Board determined this study (# 00133238) to be exempt and waived the requirement for informed consent due to the de‐identified nature of the data. The COVID‐19 Shared Data Resources were accessed and analyzed via the VA informatics and computing infrastructure server (VINCI), ensuring veterans' privacy and data security.

## Consent

The Institutional Review Board determined this study (# 00133238) to be exempt and waived the requirement for informed consent.

## Conflicts of Interest

The authors declare no conflicts of interest.

## Supporting information



Supporting Information

## Data Availability

The data that support the findings of this study are available from the US Department of Veterans Affairs Shared COVID‐19 Data Resources. Veterans Affairs (VA) data are freely available to researchers behind the VA firewall with an approved VA study protocol. For more information, visit https://www.virec.research.va.gov or contact the VA Information Resource Center at VIReC@va.gov.
