# Supplementary material for: Antihypertensive Drug Use and COVID‐19 Disease Severity in Hospitalized US Veterans: A Retrospective Cohort Study
Source: J Clin Hypertens (Greenwich). 2025 Feb 24;27(2):e70021. doi: 10.1111/jch.70021 (PMC11850436; doi:10.1111/jch.70021)
Supplement: Supplementary file 1 — Supporting Information [file JCH-27-e70021-s001.docx]

**Supplemental Table 1**: *Univariable and Multivariable Cox regression survival analyses of in-hospital COVID-19 and hypertension-positive hospitalized veterans for ventilation and fatality.We showallmodel covariates in this table, apart from hypertensive medications which are displayed in Table 2. This was done to make Table 2 succinct in the main manuscript.*

|  | Ventilation | | | | Fatality | | | |
| --- | --- | --- | --- | --- | --- | --- | --- | --- |
|  | **Univariable** | | **Multivariable** | | **Univariable** | | **Multivariable** | |
|  | **HR (95% CI)** | **p–value** | **HR (95% CI)** | **p–value** | **HR (95% CI)** | **p–value** | **HR (95% CI)** | **p–value** |
| Age, years | | | | | | | | |
| 18 – 29 | NA | NA | NA | 0.97 | NA | NA | NA | 0.97 |
| 30 – 39 | 0.54 (0.42-0.71) | **<0.001** | 0.85 (0.65-1.11) | 0.23 | 0.14 (0.09-0.23) | **<0.001** | 0.21 (0.13-0.35)) | **<0.001** |
| 40 – 49 | 0.67 (0.57-0.78) | **<0.001** | 0.84 (0.72-0.99) | **0.03** | 0.23 (0.18-0.3) | **<0.001** | 0.32 (0.25-0.4) | **<0.001** |
| 50 – 64 | 0.81 (0.75-0.86) | **<0.001** | 0.87 (0.82-0.94) | **<0.001** | 0.49 (0.46-0.53) | **<0.001** | 0.58 (0.53-0.62) | **<0.001** |
| 60 – 74 | 1 (ref) |  | 1 (ref) |  | 1 (ref) |  | 1 (ref) |  |
| 75 – 84 | 0.86 (0.8-0.91) | **<0.001** | 0.83 (0.78-0.89) | **<0.001** | 1.43 (1.36-1.51) | **<0.001** | 1.32 (1.25-1.4) | **<0.001** |
| 85+ | 0.46 (0.41-0.51) | **<0.001** | 0.44 (0.4-0.5)) | **<0.001** | 2.45 (2.31-2.59) | **<0.001** | 2.08 (1.95-2.21) | **<0.001** |
| Income Over Median | 0.96 (0.91-1.01) | 0.13 | 1 (0.94-1.05) | 0.88 | 0.96 (0.91-1) | 0.05 | 0.95 (0.9-0.99) | **0.02** |
| \| Male Sex \| 1.24 (1.08-1.41) \| 0.002 \| 1.36 (1.18-1.55) \| <0.001 \| 2.05 (1.77-2.36) \| <0.001 \| 1.48 (1.28-1.72) \| <0.001 \| \| --- \| --- \| --- \| --- \| --- \| --- \| --- \| --- \| --- \| | | | | | | | | |
| Race |  |  |  |  |  |  |  |  |
| Black | 1 (ref) |  | 1 (ref) |  | 1 (ref) |  | 1 (ref) |  |
| Other | 1.01(0.91-1.11) | 0.91 | 1.03 (0.93-1.14) | 0.05 | 1.3 (1.2-1.4) | **<0.001** | 1.19 (1.1-1.3) | **<0.001** |
| White | 0.92 (0.87-0.98) | **<0.001** | 0.92 (0.87-0.98) | **0.01** | 1.3 (1.2-1.3) | **<0.001** | 1.12 (1.06-1.18) | **<0.001** |
| BMI (kg/m^2^) | | | | | | | | |
| Underweight (< 18.5) | 0.95 (0.8-1.14) | 0.6 | 1.09 (0.91-1.3) | 0.36 | 1.49 (1.35-1.65) | **<0.001** | 1.5 (1.36-1.66) | **<0.001** |
| Normal weight (18.5 – 24.9) | 1 (ref) |  | 1 (ref) |  | 1 (ref) |  | 1 (ref) |  |
| Overweight (25 – 29.9) | 1.28 (1.19-1.38) | **<0.001** | 1.11 (1.03-1.19) | **0.01** | 0.76 (0.72-0.8)) | **<0.001** | 0.83 (0.79-0.88) | **<0.001** |
| Obese (30 – 39.9) | 1.45 (1.35-1.56) | **<0.001** | 1.1 (1.02-1.18) | **0.02** | 0.62 (0.59-0.66) | **<0.001** | 0.76 (0.72-0.81) | **<0.001** |
| Morbidly Obese (40+) | 1.71 (1.55-1.88) | **<0.001** | 1.18 (1.06-1.3) | **<0.001** | 0.58 (0.53-0.64) | **<0.001** | 0.82 (0.74-0.91) | **<0.001** |
| Smoking Status | | | | | | | | |
| Current Smoker | 0.88 (0.81-0.95) | **<0.001** | 0.94 (0.86-1.02) | 0.14 | 0.86 (0.8-0.93) | **<0.001** | 1.04 (0.96-1.12) | 0.32 |
| Former Smoker | 1.04 (0.99-1.1) | 0.13 | 1.01 (0.95-1.07) | 0.78 | 1.21 (1.15-1.27) | **<0.001** | 1.08 (1.03-1.14) | **<0.001** |
| Alcohol Dependence | 0.77 (0.72-0.83) | **<0.001** | 0.82 (0.76-0.88) | **<0.001** | 0.61 (0.57-0.65) | **<0.001** | 0.77 (0.72-0.83) | **<0.001** |
| Comorbidities | | | | | | | | |
| Asthma | 1.02(0.93-1.12) | 0.73 | 0.99(0.9-1.09) | 0.87 | 0.73(0.67-0.8) | **<0.001** | 0.83(0.76-0.92) | **<0.001** |
| CAHD | 1.01(0.96-1.06) | 0.8 | 0.92(0.87-0.98) | **0.01** | 1.31(1.25-1.36) | **<0.001** | 1(0.96-1.05) | 0.90 |
| Cancer | 1.03(0.97-1.09) | 0.33 | 1.04(0.98-1.11) | 0.2 | 1.33(1.27-1.39) | **<0.001** | 1.04(0.99-1.1) | 0.07 |
| CKD | 1.1(1.05-1.16) | **<0.001** | 1(0.94-1.07) | 0.93 | 1.55(1.48-1.62) | **<0.001** | 1.13(1.08-1.19) | **<0.001** |
| COPD | 1.06(1.01-1.12) | **0.025** | 1.01(0.96-1.08) | 0.63 | 1.2(1.15-1.26) | **<0.001** | 0.93(0.89-0.98) | **0.01** |
| Heart Failure | 1.08(1.02-1.14) | **0.008** | 0.8(0.75-0.86) | **<0.001** | 1.42(1.36-1.49) | **<0.001** | 0.94(0.89-1) | **0.04** |
| Diabetes | 1.24(1.18-1.31) | **<0.001** | 1.15(1.08-1.22) | **<0.001** | 1.1(1.05-1.15) | **<0.001** | 1.12(1.06-1.17) | **<0.001** |
| HIV | 0.88(0.67-1.14) | 0.33 | 0.94(0.72-1.23) | 0.67 | 0.62(0.47-0.81) | **<0.001** | 0.8(0.61-1.05) | 0.1 |
| Liver Disease | 1.02(0.94-1.1) | 0.60 | 0.96(0.88-1.04 | 0.28 | 0.95(0.89-1.02) | 0.18 | 1.04(0.97-1.12) | 0.23 |
| Sickle Cell | 1.06(0.66-1.7) | 0.81 | 1.02(0.63-1.65) | 0.93 | 0.94(0.6-1.45) | 0.76 | 1.07(0.69-1.67) | 0.75 |
| Charlson Comorbidity Index | | | | | | | | |
| 1 | 1 (0.91-1.11) | 0.94 | 0.98 (0.88-1.09) | 0.65 | 1.28 (1.15-1.41) | **<0.001** | 1.12 (1.01-1.25) | **0.03** |
| 2 | 1.11 (1.01-1.22) | **0.04** | 1.01 (0.91-1.12) | 0.87 | 1.47 (1.33-1.62) | **<0.001** | 1.18 (1.06-1.3) | **<0.001** |
| 3+ | 1.12 (1.03-1.21) | **0.01** | 0.92 (0.83-1.04) | 0.18 | 1.99 (1.83-2.16) | **<0.001** | 1.26 (1.13-1.4) | **<0.001** |

*Variables of significance are bolded.
